# Taming Proteus: Challenges for Risk Regulation of Powerful Digital Labor Platforms

**DOI:** 10.3390/ijerph19106196

**Published:** 2022-05-19

**Authors:** Marie Nilsen, Trond Kongsvik, Stian Antonsen

**Affiliations:** 1 Department of Industrial Economics and Technology Management, Norwegian University of Science and Technology, 7491 Trondheim, Norway; trond.kongsvik@ntnu.no (T.K.); stian.antonsen@samforsk.no (S.A.); 2 Studio Apertura, NTNU Social Research, 7491 Trondheim, Norway

**Keywords:** risk regulation, platform-mediated work, gig economy, platform economy, power, digital labor platforms

## Abstract

The platform economy’s emergence challenges the current labor regulations hinged upon the binary employer–employee relations established during the industrial age. While this burgeoning phenomenon presents several possibilities for workers, customers, and businesses alike, scholars from various fields have sounded alarms regarding pitfalls in platform-mediated work (PMW). The regulation of working conditions, health, and safety risks are integral to these worries. Drawing upon existing research and empirical data from 49 qualitative interviews with several stakeholders, this paper explores the various dimensions of power exerted by platforms and the mismatch with the current risk regulatory framework. Four regulatory gaps are identified and the concept ‘regulatory escape’ is introduced. The study posits that taming powerful platforms requires harnessing adequate regulatory capacity grounded on developing an expansive view of regulation that encompasses all forms of socio-economic influence. The paper invokes reflection on the existing regulatory systems in society and calls for a more profound and inclusive debate on platform-mediated work and how regulatory gaps can be closed.

## 1. Introduction

The convergence of technological innovation, globalization, the decline in worker representation, and the disruption of stable employment relationships are transforming the labor market and causing a deepening concern for the future of work [1,2,3]. Part of this development is the increasing participation of workers in the platform economy. Labor through digital platforms in the EU alone has been estimated to be over 28 million people, and the number is expected to increase to 43 million in 2025 [4]. Platform-mediated work (PMW) refers to paid labor provided through or mediated by a digital platform [5]. The key features of PMW include a triangular relationship between workers, platform owners, and customers and the intermediation of tasks on-demand or temporarily through online platform technology [5]. 

The essence of regulation is the protection of critical societal values by restricting some actors’ discretionary space [6,7]. Power is thus a key dimension of regulation, as authorities are assigned the power to provide limits to the power of others. Work regulation is strongly motivated by protecting workers’ safety and well-being under combined rationales based on human rights, externalities, information defects, and unequal bargaining power [8]. When it comes to platform-mediated work, issues regarding income security, access to social protection, collective bargaining, and concerns regarding occupational safety and health (OSH) have prompted national governments and policymakers at the EU level towards improving working conditions [9,10]. 

Still, aspects of PMW are hard to reach through regulatory means. For example, platform-mediated work is part of a continuous process toward the precarization of work already evident in the previous decades through offshoring and outsourcing of labor [11,12,13]. Also, digital technologies delineate platforms from traditional companies [14] in the way they transform the content, distribution, and control of work, emphasizing a break from previous approaches to labor control [15,16,17]. 

This paper focuses on identifying current regulatory challenges (the study’s focus on regulatory challenges involves limitations in that specific health-related issues or the psychological needs of workers will not be addressed in detail) related to ensuring the working conditions, health, and safety in platform-mediated work. Explicitly, we will explore the following problem formulation: *What are* *the challenges in regulating digital labor platforms in relation to working conditions and safety in platform-mediated work?* Drawing upon existing research and empirical data consisting of 49 interviews with several stakeholders, we explore the various dimensions of power exerted by platforms and the mismatch with the current regulatory framework. 

Like the oracle Proteus, a shape-shifting god in Greek mythology, platform companies are powerful entities with adaptive capabilities that evade capture. Their characteristics are hard to define in terms of existing employment and industry regulations. Despite having an arsenal of digital capabilities and extensive data, platforms share the sea god’s aversion to sharing information. The only way to capture the elusive Proteus was through careful planning and persistence. The multiplicity of labor platforms and their ability to use different dimensions of power [18] through reconfigurable features of digital technology and new business models make PMW regulation an uphill battle. Consequently, its governance must account for an array of regulatory tools and processes to address both old and new challenges while accounting for a diversity of actors, interests, and contexts encompassing its requisite variety [19]. 

The sub-section below describes platform-mediated work. The theoretical framework for this paper is divided into two sections. Section 2.1 presents PMW’s role in the growing precarity and increasing control of work through digital platform technology using Lukes’ power dimensions. Baldwin, Cave, and Lodge’s [8] concept of regulation is discussed in 2.2. The empirical basis and analysis of the data are described in Section 3. Section 4.1 briefly describes working conditions and safety in PMW based on two delivery platforms in Norway, while the subsequent Section 4.2 identifies the regulatory challenges in PMW. The final sections apply power and regulation concepts in determining ways to close the regulatory gaps. 

## 2. Background and Analytical Framework

### 2.1. Digital Labor Platforms 

Work mediated by digital platforms such as Uber, TaskRabbit, Fiverr, Upwork, and Deliveroo belongs to a polyonymous phenomenon known by a plethora of terms like *‘gig economy,’ ‘on-demand economy,’ ‘sharing economy,’ ‘collaborative economy,’ ‘platform economy,’* and *‘crowdwork’* [20,21,22]. There are two main types of work performed in PMW: online services and in-person tasks performed within a geographical area [23]. The skills required to perform labor and the task complexity occur within a broad spectrum—from a fixed-term project involving professional expertise in programming to micro-tasking [24] activities like labeling photos executed within a few minutes or one-off tasks such as house cleaning or food delivery. 

The platform economy provides several advantages: reduced transaction costs, efficient matching of supply and demand [25,26], access to affordable services [27], the unlocked value of underutilized physical assets [12], improved consumer trust through rating systems [28], additional income [22], alternative entry into the labor market [29], and flexible work scheduling [30] that provides an opportunity for combining work with care responsibilities [31]. 

The growth of digital labor platforms within Europe’s socio-economic landscape is causing several concerns that traverse various legal issues such as taxation, labor, OSH, consumer protection, GDPR, competition, and social welfare [32,33]. Critics maintain that established firms are up against platform companies who have honed their skills in playing *‘regulatory arbitrage,’* or strategies that take advantage of opportunities created by gaps in regulations [34,35,36]. There are two main issues of concern in PMW regulation: employment status and algorithmic management [37]. This twofold challenge is reflected in the two strands of research on platform labor [38]. 

PMW is a type of non-standard work [39] (i.e., not open-ended, full-time employment) where a majority of the individuals are self-employed [4]. The platforms require individuals to provide the necessary tools for production needed to accomplish the task, which platforms use to justify their worker classification [40,41]. The binary employer–employee relationship is transformed into a triadic and transient relationship between the client, the platform, and the worker. In many instances, freelancers and self-employed individuals are not covered by regulations related to the working environment, labor disputes, wage guarantee, wage during lay-off, occupational injury insurance, obligatory occupational pension, and special benefits following an occupational injury [42]. Young, self-employed workers may assume that OSH is the responsibility of the platforms and risk winding up with no protection or social safety nets [43] as they fall through regulatory gaps in the system. 

Algorithmic management is a feature that distinguishes platforms from traditional companies [14] in the manner they approach labor control [15,16,17]. Algorithmic management refers to how *”human jobs are assigned, optimized, and evaluated through algorithms and tracked data”* ([44] p. 1). The extensive work monitoring and potential biases embedded in automated decision-making systems contribute to work pressures and cloak platform accountability [45]. Calculations of supply and demand affecting the distribution of work, individual performance evaluations based on parameters often unknown to workers, and internal ranking systems that pit workers against each other in getting bonuses or preferred treatment are all incorporated into the black-boxed application (app) [15,46,47]. 

The lack of transparency around the inner workings of platform technology and the disintegration of traditional boundaries delineating organizational responsibilities pose challenges to the regulation of PMW [48]. In the following section, we will be discussing how powerful platforms and gaps in the regulation of PMW generate a mismatch between the regulator and the regulated entities.

### 2.2. Power Dimensions and Digital Labor Platforms

Globalization and the advancement of technology have resulted in more complex and stratified regulatory regimes [49]. With increasing stakeholder involvement in safety regulation [49], there is a need to examine power issues [50]. Power can be leveraged by industry over regulators who become dependent on the information companies possess [51] or harnessed by regulators to adequately match large, influential companies [52]. Power is also central to employer–employee and principal–agent relationships. Hence, examining the interface between power and regulation enables an in-depth understanding of gaps in current regulatory frameworks. More importantly, the nexus between these two concepts is key to unlocking regulatory capacities offered by a broader perception of power and regulation. The following sub-section describes digital labor platform power dimensions and the concept of regulation. This section ends with a description of the regulatory framework in the Norwegian context and the power dimensions of the regulator, the Norwegian Labor Inspection Authority (NLIA). 

To examine the power platforms have at their disposal, we adopt Lukes’ concept of power [53]. According to his three-dimensional view, it is not enough to look at power as earlier described by Dahl ([54] pp. 202–203), where “*A has power over B to the extent that he can get B to do something that B would not otherwise do.*” This one-dimensional view of power leads to an ”*exercise fallacy*” where power is restricted to observable events [55] determined by who wins the decision-making process. Another dimension, the *“second face*” of power that includes setting the agenda, was introduced by Bachrach and Baratz [56]. This two-dimensional view of power consists of the process through which individuals or groups constrain “*the scope of decision-making to relatively “safe” issues*” ([56] p. 948)—which issues are allowed to enter the decisional arena and which problems are ignored [55]. According to Lukes ([57] p. 61), an even broader view of power entails scrutinizing the more covert form of power that “*shapes desires and beliefs in the absence of observable conflict*”. Power, at its most inconspicuous, is a symbolic type of power where acquiescence is obtained by the powerful. Here, people believe to be making their own choices without actually seeing how agents advance their interest (which may also be in the interest of others) by influencing their preferences and perceptions [55,57]. 

**Figure 1:**
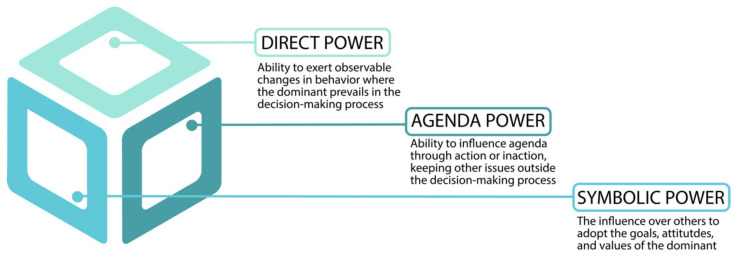
The three dimensions of power (based on [18,52]).

The three dimensions of power are illustrated in Figure 1. The first dimension, direct power, refers to the ability to exert observable changes in behavior where the dominant prevails in the decision-making process. The second dimension, agenda power, refers to the ability to influence the agenda through action or inaction. The third dimension, symbolic power, refers to the influence over others to adopt the goals, attitudes, and values of the dominant. 

#### 2.2.1. Direct Control/One-Dimensional View of Power

There are several ways in which platforms directly exercise power over other actors. One example is found in the work process design through inscribed procedures. Work is *‘“taskified*” [58] by the platform by transforming the exchange of labor for payment into standardized transactions involving atomized tasks [15]. The level of detail in which work procedures are orchestrated varies from app to app. Some platforms provide leeway for individually determining how the work is performed (such as designing a logo). In contrast, others provide stepwise instructions that constrain any further action, thus becoming ”*inescapable parts of the execution of work*” ([59] p. 14). 

Algorithmic management enables the control and monitoring of task performance comparable to a panopticon [26]. Algorithmic management profoundly constrains worker autonomy through surveillance and ranking systems that measure individual performance, customer ratings that may be used to deactivate a user’s account, and the opaqueness of how tasks are distributed [47,60,61]. Performance pressures combined with information asymmetry (see [15]) tip the power scale in favor of the platform [5].

Although platforms allow service providers to work for competing platforms, they may include lock-in mechanisms to deter workers from using other platforms. These mechanisms include penalties for rejecting or canceling tasks, non-transferable ratings, features that encourage workers to commit to the next task to avoid switching platforms, and loyalty programs that reward long hours of availability for achieving a high number of tasks per week [24,62].

#### 2.2.2. Agenda Power/Two-Dimensional View of Power

The platform’s Terms of Use (ToU) exemplify the power to set the agenda. Existing as a take-it-or-leave-it condition, users must accept the ToU to access the app and find work [63]. These unilateral conditions coerce workers into an independent contractor status [64] or impose the risk of client payment rejections and other liabilities that render workers powerless against platform companies [41,65]. Moreover, arbitrary deactivations and changes to worker piece rate with limited or no option for recourse incapacitate workers and increase emotional demands [22,66,67]. Furthermore, performance indicators serving as the yardstick for potential deactivation may not accurately reflect work efforts and may even include factors workers have no control over [48].

The deep pockets of patient venture capitalists have contributed to considerable lobbying efforts from platform behemoths like Uber and Airbnb [68,69]. Some national and supranational policymakers have been ”*dazzled by talk of innovation*” ([70] p. 3) and allowed platforms to operate without being limited by national quantitative restrictions in highly regulated sectors (e.g., transportation) under the aegis of the European single market. In setting the agenda, platforms outplay labor unions by mobilizing lobbying firms and securing meetings with relevant national and EU-level officials [70].

#### 2.2.3. Symbolic Power/Three-Dimensional View of Power

Platforms are shaping “*desires and beliefs*”*’* [55] by labeling themselves to advocate ideas promoting participation in PMW and finding ways to navigate the liminalities of regulation. 

Schumpeter’s *‘creative destruction*’ (in [71] p. 3) and ‘*disruptive technology’* ([26] pp. 31–32) are terms that often surface in the platforms’ discourse as a means to substantiate claims of innovation along with promises of work opportunities, transaction efficiency, and unlocking the value of underutilized resources [71,72]. Platforms circumvent regulatory grey areas by rebranding externalization and dissolution of responsibility as technological innovation [71]. Prassl ([26] p.31) underscores that ”*gig-economy doublespeak*” is a way for platforms to reconstruct our perceptions, not only of the industry, but also its regulation. He further points out, *”It is hard to regulate that which we don’t understand, or perceive to be novel and different”* ([26] p. 32).

Platforms cater to the narratives of empowerment, autonomy, and flexibility to entice workers to become their own boss, micro-entrepreneurs, and courier partners [22,73]. Both platforms and workers highlight the advantages of scheduling flexibility, which remains a critical point in the bandwagon for independent contractor status. The firm’s attempt to shirk away from employer responsibilities is a long-standing phenomenon, while the claim of platforms to be software companies or digital marketplaces is relatively new [15,71]. 

### 2.3. Regulation

Regulation refers to *“the use of authority (often in the hand of specialized agencies) to set and apply rules and standards*” ([74] p. 3). Before regulations can be put in place, there must be a conception of goals to be achieved by the regulation in question. Among many possible goals is the need to control or influence risk—the possibility of experiencing loss of something of value and uncertainties related to the consequences of a given activity. Therefore, risk regulation aims to avoid or control the unintended side effects of industrial activity on people’s safety, health, well-being, and the environment (see, e.g., Power, 2004). 

**Figure 2:**
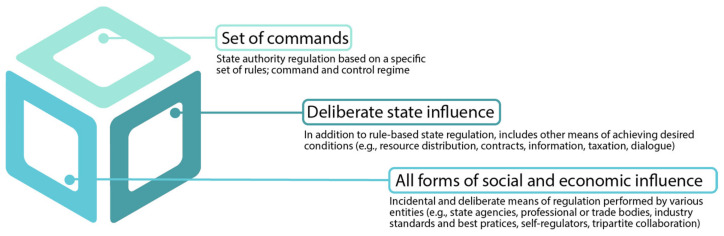
Different concepts of regulation (based on [8,52]).

The *‘use of authority’* and the *‘rules and standards’* emphasized above can have multiple meanings. Baldwin, Cave, and Lodge ([8] p. 3) identify three ways of understanding regulation (Figure 2). In the strictest sense, regulation is based on specifying a set of commands or rules by a single agency authorized for a specific purpose—the classic *‘command and control’* meaning of the term. Regulation can also be viewed as “*deliberate state influence*,” which includes other means of achieving desired conditions such as resource distribution, incentives, contractual discretion, and information dissemination, in addition to rule-based state regulations ([8] p. 3). The broadest view of regulation will include “*all forms of social or economic influence*”*’* ([8] p. 3), encompassing a wide-ranging “*use of authority*” in applying or setting the “*rules and standards*” (Hood et al., 1999:3). This comprehensive approach includes both incidental and deliberate means of regulation by various entities, from governmental agencies to trade bodies and self-regulators ([8] p. 3). 

Similar to the concept of power, regulation can be restrictive, but can also be facilitative [8,57], depending on whether or not the goals of the two parties are aligned. By bringing together the concepts of regulation and power, we can examine the interface between “*formal authority and executive power*” ([52] p. 30). Hence, we can identify strategies that may decrease the mismatch between the regulators and the platforms they regulate by examining the regulatory gaps in PMW.

### 2.4. The Norwegian Regulatory Context

The regulatory context, strategies, and power dimensions impact risk regulation [52]. Therefore, examining the regulatory context of PMW coheres with exploring the power dimensions the regulatory agency has at its disposal. To understand the mismatch between the regulator and the regulated, we must first probe into the power dimensions of the NLIA. 

#### 2.4.1. Direct Control/One-Dimensional View of Power

The Norwegian Labor Inspection Authority is the primary government agency responsible for regulating occupational safety and health in land-based operations in Norway [75]. The NLIA consists of 600 employees distributed into 16 district offices, 7 regional offices, and a directorate. From 15,265 inspections, the number decreased to 9606 in 2020 [76]. The regulatory agency’s toolbox consists of four main strategies for sanctioning violations of the Working Environment Act (WEA): orders, coercive fines, the shutdown of operations, and law enforcement involvement in serious breaches. The NLIA’s direct power based on state-vested authority over enterprise compliance (for example, see [77]) with the WEA more closely corresponds to the rule-based interpretation of the concept of regulation. 

#### 2.4.2. Agenda Power/Two-Dimensional View of Power

Concerning the Working Environment Act (WEA), NLIA’s activities include supervision, knowledge dissemination, guidance, cooperation, regulatory development, internal control audits, verifications or inspections, and accident investigations [75,76]. The Ministry of Labor determines the NLIAs goals and focus areas, including maintaining and developing working environment regulations in close collaboration with employer and employee organizations [78]. Successful tripartite cooperation is, thus, highly dependent on the quality of interaction between the parties and their degree of representation. Involving several stakeholders in the regulatory development thus empowers the different participants in co-determining the agenda related to working conditions and safety. 

#### 2.4.3. Symbolic Power/Three-Dimensional View of Power

Regulation of work in Norway is primarily based on functional regulations to underscore enterprise responsibility in systematic and continuous improvement of their safety management system [79]. This enforced self-regulation in the country incorporates tripartite collaboration or co-regulation [80]. The partnership between regulators, labor unions, and employer associations is legitimized under this expanded use of the concept of regulation [81]. Functional regulations stipulate overarching goals while leaving the enterprises to choose the specific methods to achieve those goals. Principles-based regulations (PBR) enable enterprises to work towards these goals without overly restricting their discretionary space [6,7]. Tripartite collaboration also reflects the welfare state model, a symbol of Norwegian egalitarian values and mutual trust [81,82].

From a regulatory perspective, the *‘second face’* of power, [56] agenda power, together with symbolic power or the third dimension introduced by Lukes [18], cover the broadest interpretation of the concept of regulation which includes “*all forms of social and economic influence*” [8]. Although there is no one-to-one correspondence between the power dimensions and various uses of the concept regulation, combining these two ideas contributes to a greater understanding of regulatory gaps ensuing from their intersection. 

## 3. Methodology

**Figure 3:**
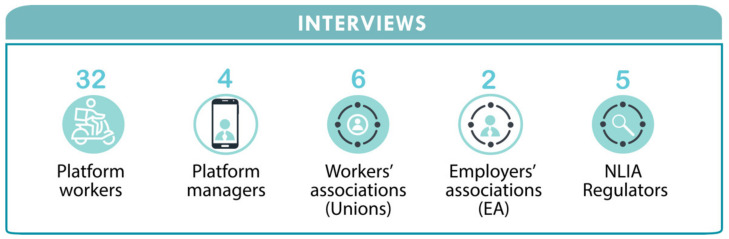
Interviews with stakeholders.

This qualitative study on the issue of risk regulation in platform-mediated labor consists of interviews from different stakeholder groups. The study adopts an interpretive approach to gain insight into the workers’ various experiences in PMW and an in-depth understanding of regulatory challenges in Norway. Semi-structured interviews were used to ensure that the essential themes were covered while also allowing for some flexibility in incorporating other issues that were important to the interviewees [83]. From a data corpus consisting of 58 interviews, the data set for this study included 49 interviews (Figure 3). 

Thirty-two platform workers from two delivery platforms with different work arrangements represent the workers’ views. In contrast, two management representatives from each platform included in this study represent the platform enterprises’ views. Six union representatives from the transport industry, two employers’ associations, and five NLIA representatives were also interviewed to represent the parties involved in the tripartite collaboration. The motivation for including a large number of platform workers compared to the other stakeholders draws attention to the reality that they are the ones who are at the receiving end of outcomes ensuing from regulatory gaps. 

The main research aims include: (a) to investigate the (mis)match between the power of labor platforms and regulator capabilities, (b) to identify potential gaps in the Norwegian context of regulating PMW, and (c) to propose potential solutions in closing the regulatory gaps. The interview guide consisted of several topics, including platform work process, working agreement and worker classification, task distribution, app features and control mechanisms (ratings, ranking systems, and others), communication channels, working conditions, health and safety, knowledge of and clarity of regulations, interaction with and role of the various stakeholders, and regulatory challenges in platform-mediated work. 

**Figure 4:**
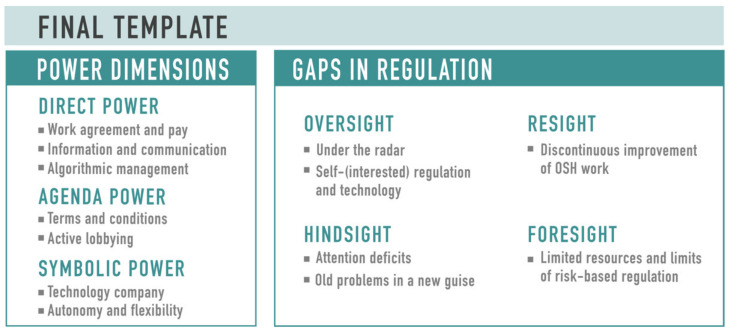
Final template for the analysis.

In analyzing the empirical data, we chose to perform a template analysis, a form of thematic analysis, described by King and Brooks [84]. The first phase involves familiarizing the empirical data through reading, re-reading, and noting ideas [85]. This is followed by the development of preliminary coding based on the selected theoretical perspectives on power and regulation, which produced six main themes explored in the concepts of power and regulation: (1) direct power, (2) agenda power, (3) symbolic power, (4) regulation as a set of commands, (5) regulation as deliberate state influence, (6) regulation as all forms of social and economic influence. The initial template was based on combining ideas from the literature on PMW, literature related to the concepts of power and regulation, and themes identified through familiarization with the data. The preliminary coding included using the NVivo search feature to find data extracts related to the codes. Data extracts (336) consisting of large chunks of text resulting from searching the data corpus were then coded and clustered into relevant themes to produce the initial template. Codes in the template were refined as the coding and analysis proceeded iteratively. Data extracts under the same code were then analyzed further to produce the final template (Figure 4). 

The final template is composed of two main categories: (1) power dimensions and (2) gaps in regulation. The former category is grouped according to Lukes’ three power dimensions and the latter is grouped into the four identified gaps in regulation. Our interview data were also cross-referenced with other data sources, such as documents provided by the workers and publicly available documents from the NLIA and the platforms.

## 4. Results

### 4.1. Working Conditions and Safety in PMW 

The two companies are global platform companies also operating in Norway. They are both registered in the SN2007 industrial code as “*53.200 Other postal and courier services*” [86]. While PC2 identifies itself as a *“delivery platform facilitating transactions*” between customers, retailers/business owners, and self-employed delivery service providers, PC1 has both employees and freelancers in its delivery fleet. Both companies require some individual investment in the equipment (e.g., vehicle, smartphone, and mobile subscription) needed for performing the delivery service. 

Unlike PC2, PC1 has safety representatives who monitor the use of helmets and ensure that lights are mounted on bikes, although self-employed workers are not covered by the WEA and are personally responsible for their own safety. There seems to be a disregard for helmet use, particularly among the increasing number of independent contractors. According to one of the PC1 couriers, “*it is just plain luck that no one has broken their skulls yet*”. Notwithstanding the same fortuity communicated by several interviewees, severe and even fatal accidents have occurred in other countries [87]. 

Seen in conjunction with incentives related to speed and a limited follow-up of helmet use, the resulting working arrangements may not be the most conducive for a high level of safety. The stark contrast between the rigorous control mechanisms embedded in apps and a more lenient attitude towards safety management is echoed by a platform worker:

*One problem with being managed by an app is that training occurs virtually, which results in many not understanding its content, that someone else can take the course for you, or that your buddy can do the job in your name, no physical checks on the equipment. It’s pandemonium combined with people who do not have a clue about their rights and duties like accident reporting.*

(PC1, Employee-17)

Platforms have several forms of power corresponding to Lukes’ three-dimensional view [18]. *Direct power* is exercised through the detailed work process inscribed in the app and piecemeal information distribution. Although the workers on both platforms can choose how to move from A to B, the interviewees concur that the app highly steers the process.

*It would be an easy job if all is well organized,* […] *it is sometimes absurd to just follow the app when it is wrong.* […] *The game is called follow the app.*

(PC1, Employee-8)

PC1 has an internal ranking system that groups the workers based on their performance. This grouping has ramifications for when they can access the available time slots and thus their opportunities for grabbing the most profitable options. PC1 workers indicated that distance is only one of the factors for distributing orders. There have been instances where they were waiting together, and only one kept getting the order. The internal ranking system determining access to future work opportunities, combined with piece-rate payment, intensifies the incentives for workers to increase delivery speed or take shortcuts.

*Sometimes, when there is traffic and I am late with the delivery, I drive past a yellow light and the police come.* […] *Yes, time pressure. There is a lot of traffic in the city center and sometimes I do not manage to be on time.*

(PC2, Freelancer-5)

The algorithm underlying the app determines work distribution. Unlike PC2, PC1 employees can request changes in some features through their union or safety representatives. Nonetheless, the potential for materializing their request may be limited since the coding occurs elsewhere, and several countries use the platform. Communication in both platforms is also limited to an in-app chat function for logistics support, e-mails, and phone (more common in PC2). This is one way platforms control the agenda; controlling communications determine which issues fall within or outside the decision-making process. Communication with fellow couriers is somewhat happenstance.

*The communication platform connecting* [workers] *directly to management has been removed; therefore, e-mails are the only point of contact.*

(PC1, Employee-14)

Another example of *agenda* *power* is requiring the user to agree to the terms of use (ToU; yes/no) set by the platform every time they use the app. The consent details are often unknown as many simply consent to access the app and the available assignments. The ToU is a powerful tool for individualizing the responsibility for safety. The interviews also revealed that changes in payment and bonus systems can also occur without workers being aware or having a say.

*They changed it without talking with me actually. Basically, if I really want, I can put them in trouble because they changed it like this. They did not contact me because my contract says* […]*. That means I make more money actually, if I use the contract. But it is good, so I am not complaining because every day I get good money and focus on myself.*

(PC2, Freelancer-8)

In using their *symbolic power*, the platforms also mirror the rhetoric described in literature around flexibility and neoliberal individualism [40,88]. The company websites attempt to attract couriers by emphasizing flexibility. This is reflected in the industry’s promotion of its interests through lobbying activities at national and supranational levels. According to the Transparency Register of the EU [89], both platforms have contacted various Commission representatives. However, the promise of flexibility is constrained by the scheduling system linked to the platform’s internal ranking system. Interviews indicate that individual responsibility for their safety is deeply ingrained among the independent contractors, despite being subject to the same control mechanisms as the employees.

[…] *you are biking, you get the same app. Almost everything is the same. The only difference is that instead of receiving a salary, you get it in the form of reversed invoicing. But I perceive it as though I am almost employed at* [PC1].

(PC1, Freelancer-8)

Management communication with the employees and with NLIA, in some ways, resembles what Prassl [26] refers to as *‘platform doublespeak’* wherein the new business model is used in arguing against requirements that do not neatly fit the enterprise. PC2 calls their workers “*courier partners*”*’* and refuses to use the words *‘shift’* or *‘turnover’* in their communication with the authors, underlining that these words imply that they are employers.

*Turnover is a term that is usually tied to permanent employment* […] *Turnover is not an important parameter when flexibility is in focus.*

(PC2, Management)

The novelty in their business model is also used to justify the impracticality of providing a physical space for employees to take breaks or interact socially with other employees. The interviews with NLIA regulators substantiate the rebranding observed in many platforms claiming that they are simply a technology company matching service providers with customers. Platform rebranding is easily verified through a search in the National Register of Business Enterprises database. In the database, the enterprises register themselves according to the industrial code (SN2007), which is based on the Nomenclature of Economic Activities (NACE Rev. 2) of the EU [86]. In the database, some digital labor platforms classify themselves as *‘programming services’* or *‘management of web portals’* instead of the main activity the platforms are involved with, i.e., delivery, cleaning, and other services [90]. In the case of delivery platforms, incorrect classification can be used to avoid requirements for affiliating the enterprise with an approved occupational health service [91]. 

### 4.2. Gaps in the Regulation

From our analysis, we have identified four types of gaps in the regulation of PMW: lack of oversight, hindsight, resight, and foresight (Figure 4). The fractures in the regulatory framework these deficiencies represent are interrelated, and their aggregation further complicates the regulation of PMW. The four regulatory lacunae in PMW are discussed in the following sub-sections.

#### 4.2.1. Lack of Oversight

The first necessary step for a regulator is having oversight of the industry–which actors are involved, what they do, and whom they employ. Therefore, this regulatory gap refers to knowledge limitations among stakeholders, conflicting goals and regulations, the inability of regulators to identify labor platforms, and a divergent platform concept of self-regulation. 

##### Under the Radar

One of the key elements in identifying which regulations are applicable is the knowledge about the entities being regulated. There is limited knowledge about the scale of platform-mediated work among the stakeholders, underpinning the difficulties scholars encounter in assessing the scale of PMW. Apart from one study conducted in 2017 [92], more current data is absent, and none of the representatives from the stakeholder groups have an overview of this emerging phenomenon in Norway. A regulator suggested that the survey’s total estimate of around 0.5% of the labor force participating in PMW may significantly underestimate reality. Thus, the inadequate evidence on the growing participation in PMW contributes to the missing impetus to address emerging concerns engendered by PMW. The report substantiates the perception that PMW is a small segment and, therefore, there is no current cause for concern.

‘*Platform doublespeak’* is operationalized in the company’s registration of industrial code. Incorrect registration contributes to the lack of a regulatory overview on PMW. The NLIA uses the industrial codes to search for companies within a specific sector and to find information on business enterprises:

*Some of them are profoundly hidden away that we do not go on inspections there. We have all registered businesses in our system, including independent contractors and companies, but it depends on what they are registered under and if the information they provide is correct. We find inconsistencies with disclosed information when we go on inspections.* […] *Often, when we go on inspections, we find out that they are not directly linked to the organization number because they are registered under several organization numbers, meaning all are independent contractors. And when they are self-employed, it becomes very difficult to see how they are linked together.*

(Regulator-5)

Representatives from employer associations and unions also share their dissatisfaction with the current system for business classification. A union representative pointed out that inconsistencies warrant reevaluating the system and the corresponding duties and responsibilities attached to the nomenclature. The industrial code may be a source of frustration for regulators. Still, according to another NLIA official, it may well be a starting point for differentiating platform economy from other businesses. 

##### Self-(Interested) Regulation and Technology

Platforms are inherent private self-regulators that unilaterally define the rules of engagement within their digital ecosystem [93]. Platforms govern through *‘hard components’* found in their platform design and through ‘*soft components’* by establishing the behavioral norms of participation in their ‘*digital space’* ([93] p. 590).

Advocates for platform self-regulation underscore government inadequacies in regulating high-speed technological development and constraints to innovation and growth [94]. The platform rationale for self-regulation, however, is based on the argument that the existing laws do not apply to them as digital platforms that only match supply and demand and, when established, threaten to close shop if regulations are not changed to their benefit ([94] p. 163). This has been observed in management discussions regarding regulatory requirements, according to an employee:

*‘Oh, we are a different kind of company. We are doing things differently; therefore, different rules apply to us.’ NO, no, no, no! Labor laws have been established for a reason, and you are trying to get around it to increase your profits.*

(PC1, Employee-7)

The platform concept of self-regulation highly departs from the idea of enforced self-regulation described above [80]. Platforms operationalize this form of self-regulation through their feedback mechanisms, terms of service, and mechanisms of enforcement [94]. The interviews with independent contractors point to unilateral decisions made by platforms in changing the bonus system and, in some cases, contracts that have grown longer and incomprehensible by the next contract-renewal round. 

#### 4.2.2. Lack of Hindsight

The benefit of hindsight offers invaluable opportunities for learning and closing the gaps in risk regulation. Accidents can result in changes to regulations and generate state or industry actions due to comprehensive media coverage and public pressure [95,96]. These ‘*focusing events’* demand attention considering the damage they inflict and can potentially inflict on society [97]. Formal and informal responses to events with dramatic impacts occur for various reasons, from providing a *“window of opportunity*” ([98] p. 650) for evaluating existing systems to demonstrating capacity for action [99] or resulting from blame avoidance [100]. Aside from events that compel immediate action, hindsight lessons learned through experience and building the capacity to address risks and manage potential occurrences systematically are thus crucial to preventing unwanted consequences. 

##### Attention Deficits

Problems concurrently compete for the limited attention capacity that decision-makers possess and are crucial in driving organizations to allocate resources to solving a particular issue [101,102]. The lack of a focusing event undermines addressing safety issues related to PMW. Unlike major accidents serving as catalysts to regulatory change [96], incidents and accidents occur independently and remain invisible to the public eye. An NLIA shares a personal reflection on the lack of momentum:

*First, development must occur; then we must say there is a problem, then we must have an opinion on how to solve the problem, then there must be political will to solve that* [PMW] *problem rather than other problems. And there are very many who have interests in the NLIA’s work, not only politicians but trade unions and large communities that have the tremendous political weight to control much of what NLIA prioritizes to do and implement.* […] *Unless there is a lot of fuss about the sharing economy, I do not think anything will happen either.*

(Regulator-3)

Although accidents in delivery work do occur, not all these events are reported. Underreporting may be partly due to the courier’s perception of safety as personal responsibility: these risks come with the job. The high threshold for reporting is also pointed out as a time-consuming activity that can decrease their delivery rate. Consequently, the high degree of underreporting also leads to the lack of data needed by regulators to support their decisions. One of the regulators pointed out that this information gap, known internally as “*no data, no problem*” hampers any dexterity to lift such issues higher up the structure.

##### Old Problems in a New Guise 

The lack of hindsight data on an outcome variable (accidents) also influences the attention to intermediate variables and root causes. The potential organizational fragmentation and negative consequences for workers outside organizational boundaries and responsibilities [48] remain unresolved. Moreover, the impact of fragmentation on working conditions and safety is potentially heightened by technology and easy labor market entry in PMW. These are organizational conditions where hindsight from other industries could provide lessons on organizational influences on safety. This was also pointed out by interviewees drawing parallels between PMW and other sectors (logistics, transport, fishery, construction, airline, and health) affected by the transfer of organizational responsibility to the individual. 

While platform managers argue that independent contractors offer flexibility in a business model focused on swift growth and expansion, workers and union representatives are concerned about the growing precarity in work-life. A courier pointed out that globalization enables strategic decisions and software development in another country. Combined with the influx of migrants looking for any type of work, it provides an opportunity for platforms to exploit.

[…] *I think there is a loophole in the law, which is harmful to society. It increases the disparities between ordinary incomes and low incomes. This means a development that is detrimental to society in the long run, so action must be taken there, politically.*

(PC1, Employee-17)

This recurring theme was also described by interviewees from the employers’ association and the union, stating that there is a need to balance worker protection and self-determination. While an interviewee (EA-2) mentioned the widespread non-compliance with maximum working hours in the legal and consulting industries, the Union representative stressed the need to protect vulnerable individuals from age-old precarity:

*There is a difference between a dog walker and a self-employed doctor. They make different choices, have different opportunities, and have different starting points. Some have to accept what they get, while others can choose and are thus also in a better negotiating position due to their expertise. I usually say that people used to stand outside factories with a hat in their hand in the old days, hoping to get a job that day. You no longer do that, but you are connected to the app, and you hope to get that job that day.*

(Union-6)

#### 4.2.3. Lack of Resight 

Regulations can be facilitative and empowering [103] through the involvement of stakeholders in the development of rules and by requiring worker participation through internal control. Successful involvement relies on establishing avenues for information sharing, problem-solving, and the development of social relations [49]. Hence, resight or the opportunity “*to get or catch sight of (someone or something) again*” [104] through formal or informal social avenues is necessary to maintain regulation’s facilitative function. Continuous improvement thus requires a level of continuity that is hard to achieve in precarious work settings like PMW.

##### Discontinuous Improvement

Regulators conduct activities using different approaches: from audits on prescriptive rules to information activities or dialogue with the industry. In a traditional work situation, the regulators identify non-compliances and instruct the company to address these problems. However, if the working relations are short-lived, the measures may be of little practical value, according to an NLIA official. 

Platform-mediated work, at least when it comes to low-skilled services, tends to be based on a highly contingent workforce and a shift from a collective to an individualized relationship between management and employees. The high level of personnel turnover involves a regulatory gap around the tripartite relationship that is a fundamental building block in regulating work-life arrangements in Nordic countries. This model rests on the assumption that there are both employers and employees and that the two parties are both able and willing to cooperate in the continuous improvement of matters of importance to working conditions.

*Getting a system to function is one thing, but people constantly leave, so one must start over frequently. You need to have good structure training. And how do you get involvement when people leave constantly? We need a new safety delegate, and the safety delegate must get training. Then we need new employees, and you don’t get continuity. Some do not care about the enterprise and just want to work and earn money. It is difficult to work towards a sound, continuous HSE system and be able to see its value along the way.*

(Regulator-5)

The gap consists of a lack of continuity among the workers in general and in critical positions of employee representation, such as safety representatives. The lack of continuity in these positions places higher demand on the platform’s investment of time and resources (regularly starting from scratch). It also changes the way regulators approach a business. This involves increased and recurring NLIA guidance on their regulation of rights and duties, the role of safety representatives, and workforce involvement by management. In many respects, the supervisory authorities will take on roles that an employer would otherwise assume. 

#### 4.2.4. Lack of Foresight

Knowledge about the characteristics and organization of an industry (oversight), its risk picture, conditions for safety (hindsight), and having the preconditions to facilitate continuous improvements (resight) are prerequisites for a proactive approach to risk and safety. Preventive strategies are essential, especially where intervention can restrain dangerous behaviors or limit severe consequences from arising [8]. The lack of information and resources may reduce the ability of regulators to deal with emerging risks within the existing strategy of risk-based regulation. 

The economic and procedural rationales surrounding the general adoption of risk-based regulation [105] can act as a roadblock to prioritizing PMW. Resource constraints impede the NLIA from prioritizing emerging risks that are less likely to gain public interest or political attention than high-potential risks or issues that permeate the public domain. The NLIA’s limited resources compared to the number of enterprises under its regulatory regime require careful selection of activities and use of resources:

[…] *we work with risk-based regulation, which means we also need to distribute the 500 inspectors across the 250,000 business entities across Norway. We also need to make hard prioritizations when we go on inspections, provide guidance, supervise, and advise. Other parts of working life are more easily accessible to us that we have a better view of, and that becomes part of our work to a greater extent.* […] *We need to know that there is a working relationship in the platform economy before we can conduct an inspection or have anything to do with that matter.*

(Regulator-2)

Another NLIA official explained that given the resource constraints, effective oversight means that their daily work aims to improve working conditions for as many as possible. In the case of PMW, where there is uncertainty in work relations between the workers and the platform company, the NLIA cannot assess individual status connected to a platform. Enforcement is an additional challenge when these platform workers are considered employees.

The regulatory supervision of PMW is exposed to a potentially self-enforcing catch-22 situation: scarce information about risks hampers resource allocation, and constrained resource allocation results in the scarcity of information about risks. If this is the case, a proactive approach to PMW is not feasible without a reactive basis of accumulated accidents providing the grounds for prioritization.

## 5. Discussion

Drawing from the empirical data, we identified four gaps in regulations encompassing inadequacies in oversight, hindsight, resight, and foresight. These flaws in the regulatory framework occur at various levels of risk regulation starting from the deficiencies existing in the organization’s composition in terms of stable structures for continuous improvement of OSH to regulatory ambiguities at the national and supranational levels. 

The heterogeneous nature of labor platforms makes it difficult to apply a one-size-fits-all approach to identifying the platforms and enforcing applicable regulations. The ambiguity surrounding the regulation of PMW lies in whether the Working Environment Act applies to all workers. Since employment status is still determined on a case-to-case basis, no deliberate state influence has so far been introduced to expedite their classification. The regulatory gaps identified in this paper are thus not likely to be easily resolved in the short term. This leaves two questions needing discussion. First, what are the drivers behind these regulatory gaps? The mechanisms creating them need to be understood in order to increase the ability to deal with such gaps. Second, how can regulatory gaps be closed? This has to do with finding ways to match the influence a regulator can have on regulated companies and the power and influence companies have over the workforce. These questions will be discussed in the remainder of the paper. 

### 5.1. The Origins of Regulatory Gaps

Regulatory gaps concerning platform-mediated work are frequently attributed to disruptive technological change revolving around information technology (e.g., [1]). However, our data indicate that the emergence of PMW is not the first example of regulatory gaps and that it is in some ways also an old problem in a new guise. For instance, Norwegian work-life arrangements were also challenged by the EU’s expansion to Eastern Europe, which quickly challenged existing agreements on minimum wage and social dumping. This is an example of a regulatory gap that does not arise from technological disruption but rather from the relationship between supranational and national regulation. Another example lies in the growth of temporary staffing recruitment agencies in the 1960s and 1970s, which was a business model growing out in response to companies’ need for numerical flexibility to meet fluctuations in demand [106]. 

The examples show that regulatory gaps are neither new nor unexpected. Regulatory gaps are a matter of lag arising from changes in the regulatory context. Developments occur swiftly, forcing regulations and regulators into a perpetual game of catch-up. The faster the rate of change, whether in terms of technology, business models, or internationalization, the more frequently we expect regulatory gaps to arise. In this perspective, the lack of hindsight, foresight, oversight, and resight associated with PMW becomes an example of a general and potentially pressing challenge to regulation and regulators’ roles and the power strategies involved in their relationship to the industries they are set to regulate. 

### 5.2. Regulatory Escape

Platform-mediated work reflects the continuing fragmentation of the organization [48] and the dissolution of legally enforced responsibility for the control of risks their operations impose. The age-old problem of misclassification and cost-reducing strategies based on the shedding of organizational responsibilities is further complicated by the platforms’ proclivity to *regulatory escape*. Both *intentional* and *incidental* types of regulatory escape can be observed in the study. Platforms may be applying strategic escape by arguing against the applicability of existing regulations and lobbying for technology- and competition-friendly policies [70], often under the guise of fair competition and innovation [107]. While the platforms in this study register themselves under the correct industrial code, a quick search in the national registry indicated that some may be taking their liberties through intentional escape. They avoid detection by classifying themselves away from existing categories that structure the attention of regulators. This can be seen as a form of arbitrage—platform companies can gain an advantage by escaping classifications (as an employer associated with a particular industry) that will subject them to health, safety, and environment (HSE) regulations and the costs of satisfying these. The power of the NLIA and the related tools at their disposal have been developed in a traditional work context where the employer is an organization and a legal entity engaging individual employees. *Direct power* in the traditional context involves that the employer is held accountable by the regulator for assuring a sound working environment and could otherwise be the subject of sanctions from the NLIA such as fines, shutdown, and law enforcement involvement. A regulatory escape from the direct power of the NLIA lies in the organization of the platform companies, where the workers are self-employed, making them responsible for their own working conditions. This is legally supported when the companies register under specific industrial codes that free them from employer responsibilities. A paradox is that the platform companies still have considerable direct power over the workers through inscribed procedures and algorithmic management [15,28].

Political priorities partly constrain the *agenda power* of regulators. In our empirical context, the Ministry of Labor determines the NLIA’s goals and focus areas annually. The prioritization of scarce resources with this as a starting point depends on information from different industries regarding issues related to safety and well-being. As interviews illustrate, underreporting of incidents, lack of focusing events, and the platform companies’ reluctance to share information make it difficult to set an agenda both on a political and regulatory level. Being partly an unintentional regulatory escape, lacking knowledge of the scope and consequences of platform-mediated work also contributes to incidental regulatory escape. 

The regulatory system in the Scandinavian countries is based on a principle of tripartite collaboration [108], where employee and employer organizations and regulators collaborate to develop functional regulations. This partnership is based on worker participation and mutual trust [49,109]. The *symbolic power* of the regulators partly involves their influence on platform companies to adopt such values. The companies also escape this form of power through the way work is organized, which repeals the possibilities for tripartite collaboration. A large portion of the workers are self-employed and are not represented by a union. Also, platform companies are primarily global enterprises following standardized business models. In some instances, they have been observed lobbying for deregulations in their countries of operation [70,110]. 

The regulatory escape can also be seen in connection with the technology underlying the platforms’ mediation of work. The algorithms connecting customers with service providers are more-or-less out of reach for supervisory authorities. They are in many ways the crown jewels of the business model, meaning that companies are likely to be wary of making them available for inspection by someone from the outside. Even if they were to be made available for supervision, the inspection would require high and specialized competence from the inspector. We see this as a form of escape due to its core role in organizing work and its power over the organization’s human side. Technology is never neutral, as values, ideas, and underlying assumptions become inscribed in technology and impact human agency [111,112]. In this case, inscription involves the atomization of work into discrete tasks matched with demand in near real-time. The atomization of the workforce, in turn, is achieved by individualizing the relationship with the company. Whether or not these embedded ideas are intentionally part of a business strategy, their consequences are palpable to those whose work is orchestrated by the app [113]. The values and social relations integrated into the platform architecture need to be examined to ensure consistency with values and goals upheld by regulations and society. Analyzing platform logic and its impact on work entails building regulatory competence in digital technology and imposing platform transparency. 

Another issue with digital platforms is their capability to gather enormous amounts of information. Zysman and Kenney [114] assert that with the amalgamation of technology and data, we need to decide who owns the data and how it can be used. Ideas, design logic, organizational processes, and strategies become progressively entangled in technology and work-life. Therefore, building technical competence needs to be prioritized so that regulatory tools and methods can adequately match contemporary work-life and provide sufficient protection to all types of workers. 

In terms of symbolic power, perhaps one must go beyond the ways labor platforms shape the narrative and perform regulatory escape. The globalized economy has changed from capitalists owning the assets to a more financialized phenomenon where good ideas gain venture capital support. Although this is beyond the scope of our paper, it is worth reflecting on what powerful institutions bet their money on and which ideas they support.

### 5.3. Could the Gaps Be Closed?

Regulation of emerging business models is clearly challenging. Historically, new regulations have been driven by public awareness of new challenges, creating political pressure to mitigate negative consequences. Regulatory authorities have limited leeway to proactively operate unless the information is available to legitimize the use of resources on emerging problems. In this subchapter, we will discuss possible measures that could support the process of closing the regulatory gaps revealed. 

#### 5.3.1. Strengthen Hindsight

The possibility of looking back offers learning opportunities. A significant hindrance to hindsight in PMW is the lack of data, including underreporting of unwanted incidents and lack of *‘focusing events’.* An underlying general cause for the lack of data is the individualization of the work and the majority of self-employed workers in PMW.

Although digital labor platforms represent nascent business models that capitalize on digital technology, they exhibit age-old problems concerning organizational responsibility. Individual consequences to platform workers often occur diffusely, unable to generate the attention necessary to induce change. Apart from a 2019 strike covered by the media [115], little has been brought to the public attention. However, recent developments at the EU level may offer favorable conditions for platform workers. Rallying cries from platform workers around the globe have captured the interest of the European Commission [116,117]. The Commission has recently submitted a proposal for a Directive on improving working conditions in platform-mediated work [118] which includes a presumption of employment status, worker rights, and increased transparency in the use of algorithms [10]. 

Adoption of the Directive provides a suitable time for reexamining existing regulations on the national level. Responding to the inadequacies may entail unconventional solutions and a broader view of regulation. Scrutiny of existing regulations should consider a more expansive baseline for worker rights, regardless of status. 

Knowledge is a critical foundation for developing relevant regulations. Acquiring “*solid evidence*” ([8] p. 310) can include novel ways of collecting accident and injury data through collaboration with platforms and government agencies and the application of Big Data. 

#### 5.3.2. Strengthen Oversight

Regulatory oversight presupposes a good overview of the area of authority. As Prassl [21] underscored, regulating something that is not understood, novel, or different, is difficult. As revealed by the interviews, the NLA lacks knowledge and oversight over the scope and content of PMW. Thus, general efforts from the regulator to seek an understanding of PMW and to monitor the industry seem to be an essential step. 

Workers for digital labor platforms are often young, and many have migrant backgrounds [22,119]. Hence, the general lack of experience and knowledge of labor regulations may need further attention from the regulatory authorities. This includes supplying easy-to-understand information and the use of various information channels and social media. 

To deter platforms from playing the game of regulatory escape, regulators could consider detaching the industrial code from OSH requirements, introducing an industrial code for digital labor platforms, or applying fines to corporations that do not register the correct industrial code. Regulations can be developed to establish the platform’s overall responsibility for OSH, similar to the Construction Client Regulations [120] and the Framework Regulations [121] in the petroleum industry. For instance, platforms can ensure that independent contractors meet company OSH standards by including the use of protective equipment in the contract. This can include requiring the platforms to provide all workers with accident insurance.

#### 5.3.3. Strengthen Foresight

Regardless of indications that PMW remains a small portion of the labor market, this labor segment will continue to grow as technology pervades the labor landscape. Risk-based regulation is based on statistics of past events and will not be sensitive to emerging risks introduced with developing technologies. More forward-facing approaches can close the gaps from inadequacies in regulation. Arenas of collaboration, including research, can uncover novel or re-emerging risks.

Regarding the forthcoming EU Directive on PMW, its implementation in Norway through the EAA will require special attention to potential deficiencies. Pulignano pointed out that the EU proposal falls short on unpaid work time and guaranteed work hours for platform workers employed by temporary agencies [122]. Bertolini et al. [123] underscored that although the EU proposal includes providing communication infrastructure for workers to “*contact and communicate with each other,”* it does little to support collective bargaining and improvement of communication between the platform and the workers. Regulators will need to continuously keep up with developments in other countries that have already implemented actions to solve issues dealing with PMW so that lessons learned from other regulatory authorities can serve as building blocks for future regulations in Norway. 

#### 5.3.4. Strengthen Resight

The *discontinuous improvement* observed in the study highlights the challenges with Internal Control and OSH activities due to a high degree of turnover. This gap is unlikely to be closed unless a paradigm change occurs. Although the NLIA is not responsible for self-employed participants in the platform economy, the resulting externalities incurred by society require attention. Thus, regulations will need to reduce incentives driving the precarization of work and provide incentives for more sustainable business strategies. 

Since tripartite collaboration remains crucial to developing relevant regulations, an arena for labor platform companies, unions, and NLIA is vital. Furthermore, stakeholders may need to consider innovative ways of revitalizing the Nordic model of tripartism and encourage platform workers to get involved in shaping the future of work.

The EU Directive on platform working conditions will most likely not be a panacea for PMW, and it is unlikely to solve other problems related to Non-Standard Employment. Since PMW is a symptom of an underlying problem, developing labor regulations to address the working conditions in PMW should incorporate improvements to working conditions of all types of work, including non-standard work arrangements. 

In closing the regulatory gaps identified in this study, the potential solutions lie in applying a broad understanding of regulation ranging from specific laws and deliberate state influence to include all forms of social and economic influence. 

## 6. Conclusions

This study has highlighted the challenges in regulating platform-mediated work. We have shown that some of these challenges lie at the core of risk regulation and supervisory activities, namely the functions we have labeled hindsight, oversight, foresight, and resight. The interrelationship between these functions might involve a self-enforcing *‘catch-22′* situation. Breaking out of this situation will require concerted action at both national and international levels. The growing interest in improving the working conditions of PMW at the EU level provides a window of opportunity to rethink how supranational and national regulations can adequately address old and new challenges and safeguard the interest of society today and in the future. Hence, it may be unwise to simply introduce ad hoc solutions designed to target the symptom. Since PMW is a manifestation of a complex underlying problem, the successful capture and taming of Proteus lies in careful consideration and firm resolve.

Amid globalization, digitalization, and profitability pressures, organizations will continue to test boundaries and maneuver through regulatory gaps. The existing regulation of work arrangements is primarily based on a dyadic relationship between employers and employees, a premise that new business models are increasingly challenging. Importantly, PMW and other forms of precarious work do not *replace* the traditional organization of working life—they come in addition to it. Although challenging, this implies that regulators should expand their toolbox to adapt to the changes in the organization of working life following a principle of requisite variety [19]. 

As organizations operate across national borders and shed off employer responsibilities, institutional safeguards must account for various contingencies while protecting individuals falling outside the binary employment model. An extensive view of regulation includes a shared responsibility among stakeholders in discussing the way forward. Regulations protecting societal values must be developed in a concerted fashion—from international conventions to local rules—to deter regulatory escape, discourage regime shopping, and close regulatory loopholes.

The transformative power of technology changes the organization of markets and how members of society interact with one another, how information flows, and the way we think. We are at a crossroads that will pave the path toward work in the near and distant future. Meaningful discourse and reflection on structuring governance systems must be considered to ensure decent and safe working conditions for all.

Further research could include exploring the effects of initiatives from supranational institutions that aim to improve the working conditions in PMW, especially the proposed directive from the EU [118]. The process of aligning supranational and national regulations is of particular interest in this context. It is also of interest to explore how the model of tripartite collaboration could be adapted to PMW, and if such collaboration could provide the regulator with a better overview of this type of work.
